# Investigation and Analysis of Genetic Diversity of Diospyros Germplasms Using SCoT Molecular Markers in Guangxi

**DOI:** 10.1371/journal.pone.0136510

**Published:** 2015-08-28

**Authors:** Libao Deng, Qingzhi Liang, Xinhua He, Cong Luo, Hu Chen, Zhenshi Qin

**Affiliations:** 1 Agricultural College of Guangxi University, Nanning 530004, China; 2 National Field Genebank for Tropical Fruit, South Subtropical Crops Research Institutes, Chinese Academy of Tropical Agricultural Sciences, Zhanjiang 524091, China; 3 Administration Committee of Guangxi Baise National Agricultural Science and Technology Zone, Baise 533612, China; 4 Guangxi Crop Genetic Improvement and Biotechnology Laboratory, Nanning 530007, China; 5 Experiment Station of Guangxi Subtropical Crop Research Institute, Chongzuo 532415, China; National Institute of Plant Genome Research (NIPGR), INDIA

## Abstract

**Background:**

Knowledge about genetic diversity and relationships among germplasms could be an invaluable aid in diospyros improvement strategies.

**Methods:**

This study was designed to analyze the genetic diversity and relationship of local and natural varieties in Guangxi Zhuang Autonomous Region of China using start codon targeted polymorphism (SCoT) markers. The accessions of 95 diospyros germplasms belonging to four species *Diospyros kaki* Thunb, *D*. *oleifera* Cheng, *D*. *kaki* var. *silverstris* Mak, and *D*. *lotus* Linn were collected from different eco-climatic zones in Guangxi and were analyzed using SCoT markers.

**Results:**

Results indicated that the accessions of 95 diospyros germplasms could be distinguished using SCoT markers, and were divided into three groups at similarity coefficient of 0.608; these germplasms that belong to the same species were clustered together; of these, the degree of genetic diversity of the natural *D*. *kaki* var. *silverstris* Mak population was richest among the four species; the geographical distance showed that the 12 natural populations of *D*. *kaki* var. *silverstris* Mak were divided into two groups at similarity coefficient of 0.19. Meanwhile, in order to further verify the stable and useful of SCoT markers in diospyros germplasms, SSR markers were also used in current research to analyze the genetic diversity and relationship in the same diospyros germplasms. Once again, majority of germplasms that belong to the same species were clustered together. Thus SCoT markers were stable and especially useful for analysis of the genetic diversity and relationship in diospyros germplasms.

**Discussion:**

The molecular characterization and diversity assessment of diospyros were very important for conservation of diospyros germplasm resources, meanwhile for diospyros improvement.

## Introduction

The genus diospyros (*Ebenaceae*) consists of approximately 400 to 500 highly heterozygous species, most of which are distributed in the subtropical and tropical regions of Asia, Africa, and central South America [1,2]. *Diospyros* Linn originated from China, and has over 900 cultivars [3,4]. Nine diospyros species are cultivated in China as fruit trees or rootstock, among these, *D*. *kaki* Thunb is the most economically valuable and widely cultivated species [5,6]. *D*. *kaki* Thunb in China accounting for 89.79% and 73.84% of total planting area and yield worldwide, respectively [7].

Guangxi is one of the most important production areas for diospyros, and diospyros germplasm resources are abundant and widely distributed because of the unique climate and diverse ecology [8]. Previous reports had mainly focused on planting cultivars, without local and natural varieties. Therefore, the assignment of cultivar identity is a major problem due to insufficient information, meanwhile, due to presence of synonyms and homonyms among local and natural varieties, which affect significantly the exploration, utilization, and protection of diospyros germplasm resources in Guangxi.

Investigation and analysis of genetic diversity of diospyros germplasms will provide information basis to support diospyros improvement and botanical research as well as support conservation efforts. So far, different marker techniques such as plant morphology [9], karyotype [10], isoenzyme [11] and DNA-based markers random amplified polymorphic DNA (RAPD) [12], simple sequence repeat (SSR) [13], restriction fragment length polymorphism (RFLP) [14], sequence-related amplified polymorphism (SRAP) [4], amplified fragment length polymorphism (AFLP) [15], and inverse sequence-tagged repeat (ISTR) [2] have been applied to study the genetic diversity and relationships between diospyros species and their relatives. Nevertheless, the relationships between diospyros accessions of local and natural varieties are still not completely clarified in spite of all previous efforts, probably because of the low resolution of the germplasm resources collection, conservation and exploitation.

Recently, a molecular marker technique termed start codon targeted (SCoT) polymorphism, a simple and novel DNA marker technique, was developed by Collard and Mackill [16]. SCoT marker technique is a simple and novel targeted molecular marker tool which base on the short conserved region flanking the start codon (ATG) in plant genes; it can generate more information related to biological traits than random DNA markers [16]. SCoT markers employ longer primers (18-mer) producing high polymorphism which is reproducible [17], it is suggested that primer length and annealing temperature are not the sole factors determining reproducibility [16]. As a single primer amplification molecular marker technique, SCoT markers are technically simple, not time-consuming and not laborious, and requires no prior sequence information and targeting functional regions [18]. SCoT markers have been broadly and successfully used for evaluation of genetic diversity, phylogenetics, fingerprinting, variation, and differentiation since 2009 [17–19].

However, up to now, no report has been available on the investigation and analysis of genetic diversity of diospyros germplasms using SCoT molecular markers in Guangxi. The objective of the present research was to identify the genetic diversity and relationships of diospyros germplasm of the local and natural varieties to provide theoretical basis for classification, protection, and utilization of diospyros germplasm resources in Guangxi China.

## Materials and Methods

### Plant material

**Fig 1:**
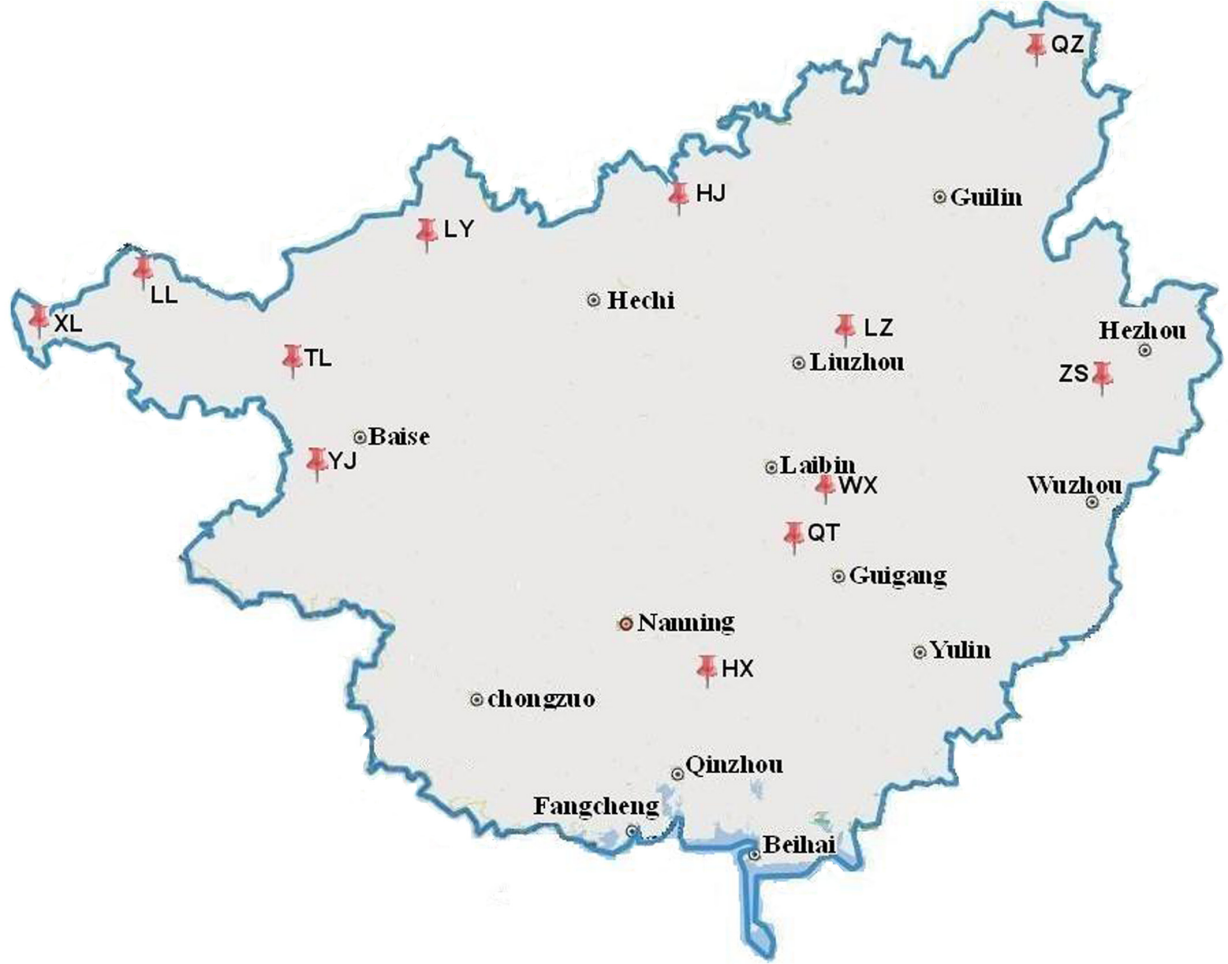
The localities of the natural *D*. *kaki var*. *silvestris* Mak samples.

A total of 95 *Diospyros* Linn and 189 *D*. *kaki* var. *silvestris* individuals corresponding to 12 natural populations were investigated and labeled in Guangxi Zhuang autonomous region, China (Fig 1, Table 1, and S1 Table), then young leaves were collected from the labeled individuals in the wild area, frozen in liquid nitrogen, and stored at -80°C until analysis.

**Table 1:** Diospyros germplasm resources and their respective localities.

NO.	Germplasms	Species	Localities	NO.	Germplasms	Species	Localities
1	Yueshi	*D*. *kaki* Thunb	Gongcheng county	49	PL1	*D*. *oleifera* Cheng	Pingle county
2	Niuxinshi	*D*. *kaki* Thunb	Pingle county	50	PL2	*D*. *oleifera* Cheng	Pingle county
3	Jixinshi	*D*. *kaki* Thunb	Longlin county	51	JC1	*D*. *oleifera* Cheng	Jinchengjiang district
4	Xiaofangshi	*D*. *kaki* Thunb	Leye county	52	JC2	*D*. *oleifera* Chen	Jinchengjiang district
5	Dafangshi	*D*. *kaki* Thunb	Leye county	53	YZ1	*D*. *oleifera* Cheng	Yizhou city
6	Huoshi	*D*. *kaki* Thunb	Gongcheng county	54	YZ2	*D*. *oleifera* Cheng	Yizhou city
7	Jingshi	*D*. *kaki* Thunb	Hengxian county	55	YZ3	*D*. *oleifera* Cheng	Yizhou city
8	HZ	*D*. *kaki* Thunb	Zhongshan county	56	YZ4	*D*. *kaki* Thunb	Yizhou city
9	LQ1	*D*. *kaki* Thunb	Liangqing district	57	YZ5	*D*. *oleifera* Cheng	Yizhou city
10	LQ2	*D*. *kaki* Thunb	Liangqing district	58	YZ6	*D*. *oleifera* Cheng	Yizhou city
11	LL1	*D*. *oleifera* Cheng	Longlin county	59	YZ7	*D*. *oleifera* Cheng	Yizhou city
12	LL2	*D*. *kaki* Thunb	Longlin county	60	YZ8	*D*. *oleifera* Cheng	Yizhou city
13	LL3	*D*. *kaki* Thunb	Longlin county	61	YZ9	*D*. *oleifera* Cheng	Yizhou city
14	LL4	*D*. *kaki* Thunb	Longlin county	62	YZ10	*D*. *oleifera* Cheng	Yizhou city
15	LY1	*D*. *kaki* Thunb	Leye county	63	YZ11	*D*. *kaki Thunb*	Yizhou city
16	LY2	*D*. *kaki* Thunb	Leye county	64	YZ12	*D*. *kaki* Thunb	Yizhou city
17	LY3	*D*. *kaki* Thunb	Leye county	65	YZ13	*D*. *kaki* Thunb	Yizhou city
18	LY4	*D*. *kaki* Thunb	Leye county	66	QZ1	*D*. *oleifera* Cheng	Quanzhou county
19	LY5	*D*. *oleifera* Cheng	Leye county	67	QZ2	*D*. *oleifera* Cheng	Quanzhou county
20	LY6	*D*. *oleifera* Cheng	Leye county	68	QZ3	*D*. *oleifera* Cheng	Quanzhou county
21	LY7	*D*. *oleifera* Cheng	Leye county	69	QZ4	*D*. *oleifera* Cheng	Quanzhou county
22	LY8	*D*. *oleifera* Cheng	Leye county	70	QZ5	*D*. *oleifera* Cheng	Quanzhou county
23	GN1	*D*. kaki Thunb	Gangnan district	71	QZ6	*D*. *oleifera* Cheng	Quanzhou county
24	GN2	*D*. kaki Thunb	Gangnan district	72	QZ7	*D*. *oleifera* Cheng	Quanzhou county
25	GN3	*D*. kaki Thunb	Gangnan district	73	QZ8	*D*. *oleifera* Cheng	Quanzhou county
26	GN4	*D*. *kaki* Thunb	Gangnan district	74	QZ9	*D*. *oleifera* Cheng	Quanzhou county
27	GN5	*D*. *kaki* Thunb	Gangnan district	75	QZ10	*D*. *oleifera* Cheng	Quanzhou county
28	GB1	*D*. *kaki* Thunb	Gangbei district	76	XL1	*D*. *oleifera* Cheng	Xilin county
29	GB2	*D*. *kaki* Thunb	Gangbei district	77	XL2	*D*. *oleifera* Cheng	Xilin county
30	GB3	*D*. *kak i*Thunb	Gangbei district	78	XL3	*D*. *oleifera* Cheng	Xilin county
31	GB4	*D*. *kak i*Thunb	Gangbei district	79	XL4	*D*. *oleifera* Cheng	Xilin county
32	GB5	*D*. *kaki* Thunb	Gangbei district	80	XL5	*D*. *kaki* Thunb	Xilin county
33	GB6	*D*. *kaki* Thunb	Gangbei district	81	XL6	*D*. *oleifera* Cheng	Xilin county
34	GB7	*D*. *kaki* Thunb	Gangbei district	82	XL7	*D*. *oleifera* Cheng	Xilin county
35	GB8	*D*. *kaki* Thunb	Gangbei district	83	XL8	*D*. *oleifera* Cheng	Xilin county
36	GB9	*D*. *oleifera* Cheng	Gangbei district	84	XL9	*D*. *oleifera* Cheng	Xilin county
37	GB10	*D*. *oleifera* Cheng	Gangbei district	85	XL10	*D*. *oleifera* Cheng	Xilin county
38	GB11	*D*. *oleifera* Cheng	Gangbei district	86	TL1	*D*. *oleifera* Cheng	Tianilin county
39	GB12	*D*. *oleifera* Cheng	Gangbei district	87	TL2	*D*. *oleifera* Cheng	Tianilin county
40	QB1	*D*. *oleifera* Cheng	Qinbei district	88	TL3	*D*. *oleifera* Cheng	Tianilin county
41	QB2	*D*. *oleifera* Cheng	Qinbei district	89	WX	*D*. *oleifera* Cheng	Laibin county
42	QB3	*D*. *oleifera* Cheng	Qinbei district	90	Yeshi1	*D*.*kaki var*.silvestris	Pingle county
43	QB4	*D*. *kaki* Thunb	Qinbei district	91	Yeshi2	*D*.*kaki var*.silvestris	Pingle county
44	QB5	*D*. *oleifera* Cheng	Qinbei district	92	Yeshi3	*D*.*kaki var*.silvestris	Leye county
45	QB6	*D*. *oleifera* Cheng	Qinbei district	93	Yeshi4	*D*.*kaki var*.silvestris	Tianlin county
46	GC1	*D*. *oleifera* Cheng	Gongcheng county	94	Yeshi5	*D*.*kaki var*.silvestris	Quanzhou county
47	GC2	*D*. *oleifera* Cheng	Gongcheng county	95	Junqianzi	*D*.*lotus* Linn	Gongcheng county
48	GC3	*D*. *oleifera* Cheng	Gongcheng county				

Note: The germplasms that have not been previously reported, or whose names are not sure were temporarily named after the first letter of their localities and numbers.

### Ethics Statement

The study was approved by Guangxi Zhuang autonomous region government, which was part of diospyros germplasm resources protection in China and supported by the public basic scientific research project foundation of Guangxi Zhuang Autonomous Region (Rzz200901).

### DNA extraction and genotype analysis

DNA was individually extracted as described by [20–22]. PCR reactions were performed in 20 μL volumes containing 2.0 μL of 10× buffer, 30 ng of genomic DNA, 30 μmol·L^-1^ primers, 4.0 mmol·L^-1^ dNTPs, and 0.2 unit of *Taq* polymerase (BoRi, Hangzhou, China). SCoT-PCR Amplification was performed in a GeneAmp PCR System 9700 (Perkin-Elmer Corp., Norwalk, CT, USA) under initial denaturation for 4 min at 95°C, followed by 36 cycles of 40 s at 95°C, 40 s at 50°C and 2 min at 72°C, followed by final extension of 7 min at 72°C.

SSR primers were also used to analyze and verify the genetic diversity and relationship of local and natural varieties in Guangxi Zhuang Autonomous Region of China, which were previously described in detail [22–24]. SSR-PCR Amplification was performed under initial denaturation for 5 min at 94°C, followed by 30 cycles of 50 s at 94°C, 1 min at annealing temperature and 50s at 72°C, followed by final extension of 4 min at 72°C. The SCoT-PCR and SSR-PCR products were separated and stained, following [25].

### Data analysis

The SCoT and SSR profiles for each band were scored as present (1) or absent (0) based on size comparison with the standard (2000 bp DNA Ladder Plus). Cluster analysis and dendrogram construction were carried out using NTSYS-pc 2.1e software [26]. Cluster analysis based on the genetic similarity matrix was performed using unweighted pair group method with arithmetic mean (UPGMA) method [27]. Nei’ s gene diversity index (He), Shannon’s information index (Ho), observed number of alleles (Na), and effective number of alleles (Ne) were calculated using the GenAlEx 6.5 program [28]. Nei’s unbiased genetic distances were calculated for all population pairs, and were used to construct a phylogenetic tree [29]. Principal coordinate analysis (PCoA) [30] was performed based on the variance covariance matrix calculated from the marker data. The distribution of genetic variation among the populations was analyzed using AMOVA 1.55 software, and a Mantel test in NTSYS-pc 2.10e software was used to test whether the matrix of the genetic distances correlates with the matrix of geographical distances.

## Results

### Analysis of validation, amplification and polymorphic of SCoT markers in diospyros germplasm resources

A total of 80 pairs of SCoT primers were selected from the previous literatures [16,17], which were used to screen for polymorphic markers using three accessions (S2 and S3 Tables) and the polymorphic markers identified were used to genotype individuals of 95 *Diospyros* Linn and 189 *D*. *kaki* var. *silvestris*. Only primers that exhibited unambiguous and reproducible band patterns were selected for further analysis. Thus, a total of 18 primers that exhibit distinct and reliable band patterns were utilized for bands scoring.

Genotypes of 95 diospyros germplasm Accessions were analyzed using the 18 screened primers. Up to 241 unambiguous and reproducible bands were generated, of those, 233 (96.68%) were polymorphic; only eight fragments were present in all the 95 accessions. The sizes of the amplified bands ranged from 250 bp to 2500 bp, of those, most of the band sizes ranged from 500 bp to 2000 bp (S1 Fig). The number of bands amplified by the selected primers ranged from 7 to 16 per primer, with an average of 13.39 bands per primer.

### Genetic diversity analysis of diospyros germplasm resources using SCoT markers

The coefficients of similarity between the germplasms were calculated using Ntedit software. These results showed that the lowest similarity coefficient was observed between YZ4 and QZ4, which was 0.485. The highest similarity coefficient was observed between GN3 and GB7, which was 0.946. The data indicated that the genetic relationship between YZ4 and QZ4 was the farthest while the genetic relationship between GN3 and GB7 was the nearest.

**Fig 2:**
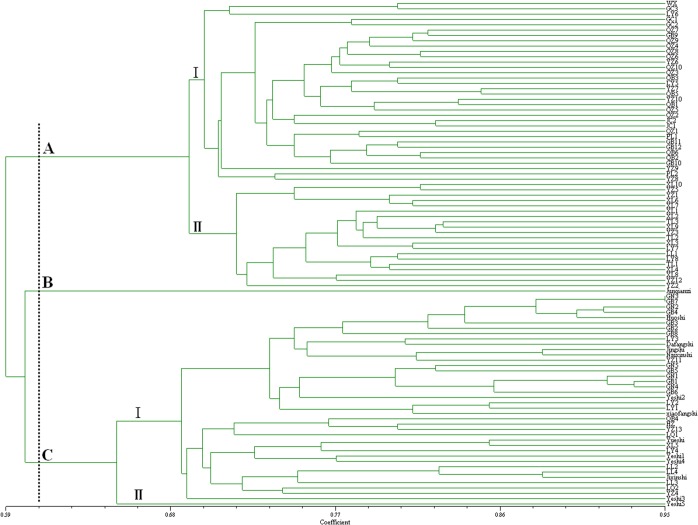
UPGMA dendrogram of the accessions of 95 diospyros germplasms based on SCoT molecular markers.

Based on corresponding similarity coefficients among the tested accessions of 95 diospyros germplasms, a dendrogram was constructed (Fig 2). The cluster analysis demonstrated that the genetic relationship of the diospyros germplasms accessions genotypes was complex. The 95 accessions fell under divided into three major groups at a genetic distance threshold of 0.608. Group A included 54 accessions, all of which belonged to *D*. *oleifera* Cheng. Group B contained only one accession belonged to *D*. *lotus* Linn. Group C included 40 accessions, 35 of those belonged to *Diospyros kaki* Thunb and 5 belonged to *D*. *kaki var*. *silvestris* Mak. These results indicated that *D*. *kaki* Thunb, *D*. *oleifera* Cheng, and *D*. *lotus Linn* could be distinguished from each other by the18 SCoT primers selected, and the genetic relationship of *D*. *kaki* Thunb and *D*. *kaki var*. *silverstris* Mak seemed closer than those of other species.

### Principal component Analysis (PCA) based on the genetic similarity matrix of SCoT

Principal component Analysis (PCA) based on the genetic similarity matrix of SCoT was conducted and the two-dimensional clustering was obtained (S2 Fig), 95 accessions of diospyros germplasms were divided into four group, the accessions of group A belonged to *D*. *oleifera* Cheng, group B belonged to *D*. *lotus* Linn, group C belonged to *Diospyros kaki* Thunb and *D*. *kaki var*. *silverstris* Mak, and group D belonged to *D*. *kaki var*. *silverstris* Mak only. The result of principal component analysis was corresponding to clustering analysis.

### Genetic diversity analysis of diospyros germplasm resources using SSR markers

In order to further verify the stable and useful of SCoT markers in diospyros germplasms, 15 SSR markers screened that showed polymorphisms selected from previous research were also used to analyze the genetic diversity and relationship in the same diospyros germplasms accessions. Results indicated 159 unambiguous and reproducible bands were generated, of those, 154 were polymorphic. The number of bands amplified by the selected primers ranged from 7 to 14 per primer, with an average of 10.60 bands per primer. The sizes of the amplified bands ranged from 80 bp to 500 bp, of those, most of the band sizes rangeed from 80 bp to 230 bp (S3 Fig and S4 Table). The coefficients of similarity between the germplasms were also calculated using Ntedit software, once again, results showed that the lowest similarity coefficient was observed between YZ4 and QZ4.

**Fig 3:**
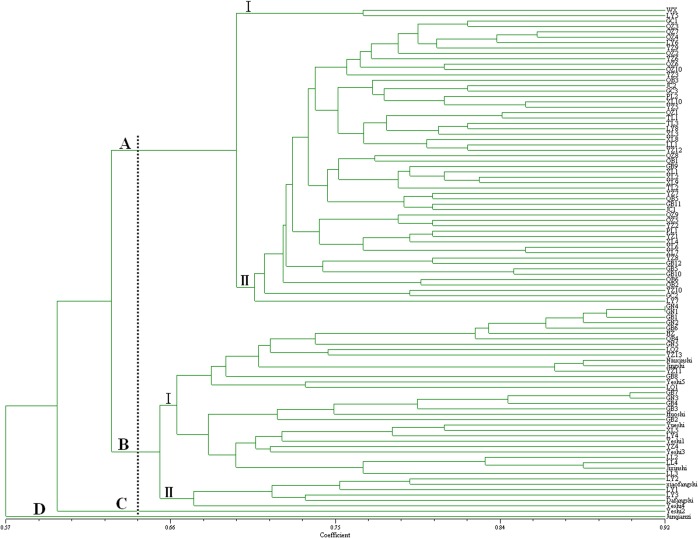
UPGMA dendrogram of the accessions of 95 diospyros germplasms based on SSR molecular markers.

A dendrogram analyzed using SSR markers was also constructed (Fig 3). The 95 accessions fell under divided into four major groups at a genetic distance threshold of 0.642. Group A contained 55 accessions of D. oleifera Cheng, Group B contained 34 accessions of *Diospyros kaki* Thunb and 5 accessions of *D*. *kaki var*. *silverstris* Mak. Group C contained only one accession belonged to *D*. *kaki var*. *silverstris* Mak. Group D contained one accession belonged to *D*. *lotus* Linn. These results indicated that *D*. *kaki* Thunb, *D*. *oleifera* Cheng, and *D*. *lotus* Linn could be distinguished from each other by the15 SSR primers selected, group A belonged to *D*. *oleifera* Cheng, group B belonged to *Diospyros kaki* Thunb and Group B belonged to *D*. *lotus* Linn. The genetic relationship of *D*. *kaki* Thunb and *D*. *kaki var*. *silverstris* Mak seemed closer than that of other species; however, genetic variation between wild persimmon germplasm belonged to different sources was larger. Nevertheless, the results of SSR molecular markers were basically consistent with that of molecular markers SCoT analysis.

Principal component Analysis (PCA) based on the genetic similarity matrix of SSR analysis was conducted and the two-dimensional clustering was obtained (S4 Fig), 95 accessions of diospyros germplasms were divided into four group, the accessions of group A belonged to *D*. *oleifera* Cheng, group B belonged to *Diospyros kaki* Thunb and *D*. *kaki var*. *silverstris* Mak, group C belonged to *D*. *kaki var*. *silverstris* Mak and group D belonged to *D*. *lotus* Linn. The results of principal component analysis were corresponding to clustering analysis.

### Genetic diversity analysis of natural *D*. *kaki var*. *silverstris* Mak populations

The genotypes of 189 individuals from 12 natural *D*. *kaki var*. *silverstris* Mak populations were analyzed using 13 selected primers. Altogether, 159 unambiguous and reproducible bands were generated, of those, 155 (97.48%) were polymorphic, with sizes ranging from 300 bp to 1800 bp (S3 Table). The number of bands ranged from 9 to 17 per primer, with an average of 12.23 bands per primer. These results showed that the natural *D*. *kaki* var. *silverstris* Mak distributed in Guangxi were rich in polymorphisms.

The genetic diversity among Zhongshan populations was determined to be the highest, with He and Ho values of 0.178 and 0.269, respectively. By contrast, the genetic diversity among Youjiang populations was found to be the lowest, with He and Ho values of 0.122 and 0.192, respectively. The genetic diversity values of these populations were ranked as Zhongshan > Leye > Luzhai > Quanzhou > Tianlin > Xilin > Hengxian > Longlin > Qintang > Wuxuan > Huanjiang > Youjiang from high to low. The number of alleles (Na) and effective number of alleles (Ne) showed the same results (Table 2).

**Table 2:** Results of analysis of the genetic diversity of the 12 natural *D*. *kaki* var. *silverstris* Mak. populations.

Population	size	Observed number of allele/Na	Effective number of allele/Ne	Shannon’s information index/Ho	Nei’s gene diversity/He	Percentage of polymorphic loci (PPL)/%
LL	16.000	1.528	1.224	0.239	0.146	64.15
XL	15.000	1.226	1.247	0.233	0.150	53.46
TL	19.000	1.415	1.258	0.247	0.157	62.89
YJ	15.000	1.239	1.199	0.192	0.122	48.43
QZ	14.000	1.201	1.278	0.240	0.160	49.69
HJ	16.000	1.170	1.218	0.196	0.128	45.28
ZS	14.000	1.321	1.307	0.269	0.178	57.23
LZ	16.000	1.277	1.272	0.245	0.160	57.23
WX	15.000	1.164	1.224	0.201	0.131	48.43
QT	18.000	1.289	1.232	0.222	0.142	54.09
LQ	16.000	1.333	1.249	0.232	0.149	57.86
LY	15.000	1.434	1.260	0.257	0.162	63.52
mean	15.750	1.300	1.247	0.231	0.149	55.19
total	189.000	1.975	1.424	0.417	0.266	97.48

Data from the AMOVA molecular detection prove that inter-populations have 54.12% genetic variation (Table 3), the results showed that the genetic differentiation of *D*. *kaki* var. *silverstris* Mak was more highly affected in inter-population groups. Gene flow, estimated by Gst [Nm = 0.5 (1—Gst)/Gst)], was 0.585, indicated that genetic differentiation exists between the natural *D*. *kaki* var. *silverstris* Mak populations and that species variation was also affected within the population. The SCoT data were analyzed through PCoA. The accumulative contribution rate of the first and second coordinates was 49.96%, indicated the main original information. The 189 individuals in the two-dimension diagram occupy a large space and were widely distributed, showed that its genetic background was broad and extremely diverse. Some populations overlap and interact, showed that the permeation of genes and communication took place among the natural *D*. *kaki* var. *silverstris* Mak populations (S5 Fig). The geographic distance and genetic distance between the wild *D*. *kaki* var. *silverstris* Mak populations were calculated through Mantel test analysis, results showed that genetic distance had positive relationship with geographical distance (*r* = 0.5559; S5 Table).

**Table 3:** Analysis of molecular variance (AMOVA) within and among natural *D*. *kaki* var. *silvestris* Mak. populations.

Source of variance	d.f	SSD	MSD	Variance component	Percentage(%)	P*
Among populations (AP)	11	2198.555	199.869	11.814	45.88	< 0.001
Within populations (WP)	177	2466.979	13.938	13.938	54.12	< 0.001
Total	188	4665.534				

Note: MSD,expected mean squares

*Number of permutation = 1,000

**Fig 4:**
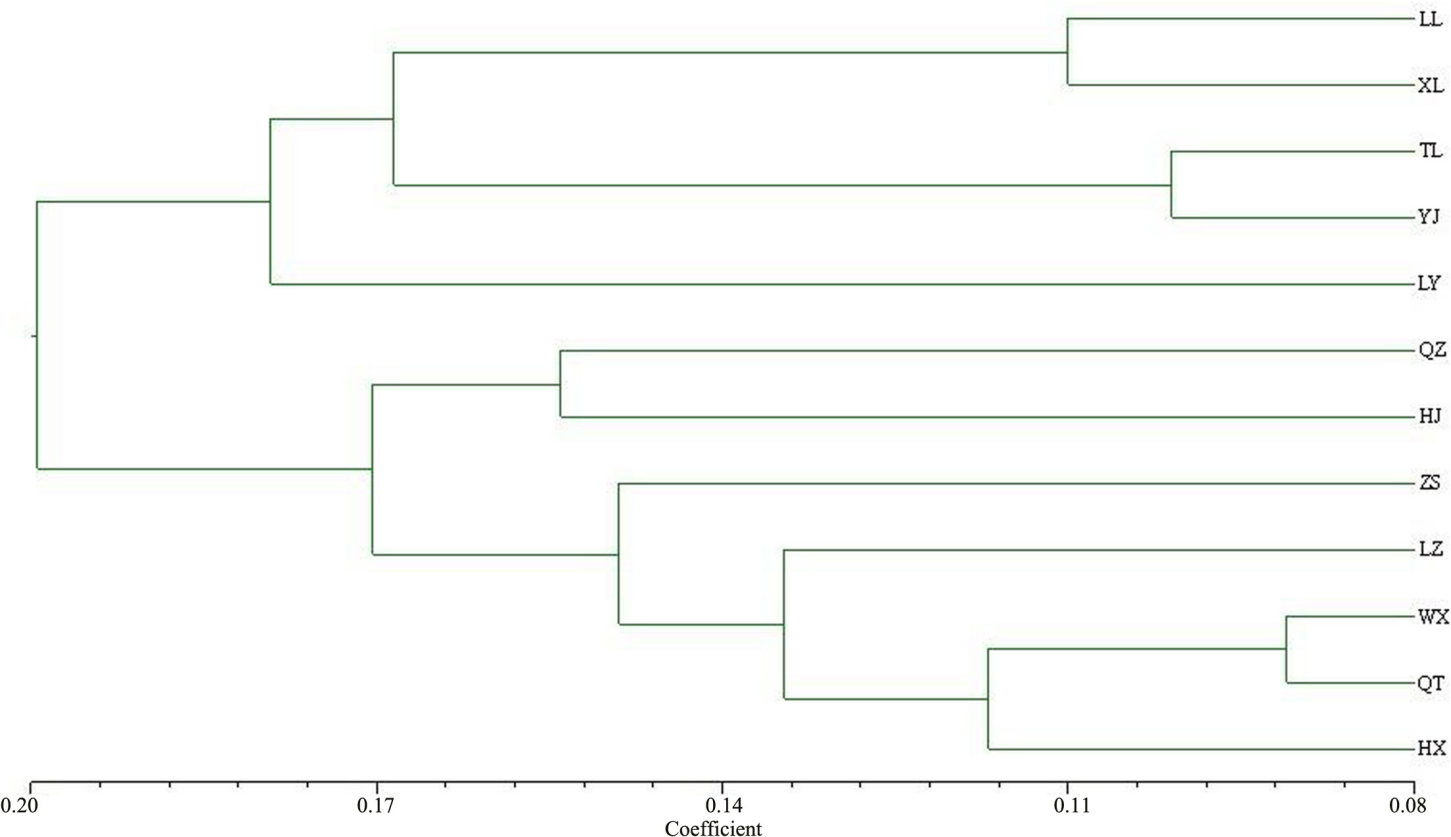
UPGMA dendrogram of the natural *D*. *kaki var*. *silvestris* Mak populations based on Nei’s genetic distance.

A dendrogram was constructed based on the results of Nei’s genetic distance using the UPGMA method (Fig 4). Twelve natural *D*. *kaki* var. *silverstris* Mak populations in Guangxi were divided into two clusters at similarity coefficient of 0.19. One cluster contained populations from Longlin, Xilin, Tianlin, Youjiang, and Leye; whereas the other contained populations from Quanzhou, Huanjiang, Zhongshan, Luzhai, Wuxuan, Qintang, and Hengxian. The genetic distance between the Wuxuan and Qintang populations was 0.091, and the genetic relationship was the closest among the populations. However, the genetic distance between the Youjiang and Quanzhou populations was 0.292, showed the farthest genetic distance. The clustering result supported the distribution of the two principal coordinates by PCoA.

## Discussion

Guangxi is located in low latitude ranging from 50 m to 1500 m. diospyros has been cultivated in Guangxi for over 400 years, and possesses abundant germplasm resources. The diospyros germplasm resources in Guangxi are not fully utilized because of poor identification and insufficient information. Many diospyros germplasms around the region that had low commodity value were neglected, and were usually cut down or top grafted by local farmers. The genetic diversity of diospyros germplasm resources decreased with the passage of time, and many rural local varieties that had great value in germplasm preservation and breeding died due to the lack of protection. Most diospyros germplasms in China were local varieties, meanwhile, another diospyros germplasms were developed through bud mutation, nevertheless, which were sporadic [3,31]. Thus, it is necessary to investigate and analyze the genetic diversity of diospyros germplasms to provide theoretical basis for classification, protection and utilization of diospyros germplasm resources in Guangxi China.

Molecular marker technology has been widely applied in research on diospyros germplasm resources [13,32,33]. Some studies suggested that the genetic diversity of diospyros germplasms revealed by SCoT markers was consistent with phenotypic analysis [34]. *D*. *kaki* Thunb, *D*. *oleifera* Cheng, and *D*. *lotus* Linn germplasms were used in current experiment to be completely distinguishable through UPGMA analysis. The *D*. *kaki* var. *silvestris* Mak germplasms are closer to *D*. *kaki* Thunb than the other species. *D*. *kaki* var. *silvestris* Mak is the native species of *D*. *kaki* Thunb [35]. Meanwhile, in order to further verify the stable and useful of SCoT markers in diospyros germplasms, SSR markers were also used in current research to analyze the genetic diversity and relationship in the same diospyros germplasms. The results of SSR molecular markers were basically consistent with that of molecular markers SCoT analysis. Thus SCoT markers were stable and especially useful for analysis of the genetic diversity and relationship in diospyros germplasms.

The climate in Guangxi greatly varies from the south to the north, but various natural *D*.*kaki* var. *silvestris* Mak populations from different regions were clustered together. Twelve populations separately gather into two big clusters. One cluster includes populations from Leye, Xilin, Tianlin, and Youjiang, which were in northwestern Guangxi, the part of Yunnan-Guizhou plateau with mountainous terrain and high altitude. The other clusters included Quanzhou of northeastern Guangxi, Huanjiang of northern Guangxi, Zhongshan of eastern Guangxi, Luzai, Wuxuan, and Qintang of central Guangxi, and Hengxian of southern Guangxi. Population genetic structures were often influenced by many factors, including habitat fragmentation, gene flow, breeding system, and seed dispersal mechanism [36,37]. When gene flow (Nm) < l, the population was more susceptible to genetic drift [38,39]. The average Nm value of natural *D*. *kaki* var. *silvestris* Mak is 0.585, which indicated that gene exchange between the populations was restricted, and the genetic differentiation in each population was severe.

The level of population genetic diversity was related to the size of the population; bigger populations generally have higher levels of genetic diversity [39]. The results of current study indicated that the genetic diversity of the Zhongshan population was the highest and that of the Youjiang population was the lowest. These results showed that the habitats of natural *D*. *kaki* var. *silvestris* Mak populations in Youjiang, Huanjiang, Wuxuan, and Qintang had been seriously damaged, and that the number of individuals has reduced.

In conclusion, SCoT molecular markers help assess genetic diversity among accessions or natural populations from different areas. The diospyros germplasm resources in Guangxi possess broad genetic background and have rich diversity, but some rural varieties and natural populations are in danger of being lost. Effective measures to protect the genetic diversity of diospyros germplasm resources are necessary. These results of current research will help in the classification, preservation, and utilization of diospyros resources.

## Supporting Information

S1 Fig Amplification generated by SCoT49, with part of the diospyros germplasm DNA templates. (TIF)

S2 Fig Principal coordinate analysis of 95 accessions of diospyros germplasms based on SCoT analysis. (TIF)

S3 Fig Amplification generated by SSR30, with part of the diospyros germplasm DNA templates. (TIF)

S4 Fig Principal coordinate analysis of 95 accessions of diospyros germplasms based on SSR analysis. (TIF)

S5 Fig Scatter diagram based on the first two principal coordinates by principal coordinate analysis (PCoA). (TIF)

S1 Table The surveyed populations and their respective ecological and geographical parameters. (DOC)

S2 Table SCoT primers used in the diversity analysis of different diospyros germplasms. (DOC)

S3 Table SCoT primers used in the diversity analysis of natural populations of *D*. *kaki var*. *silverstris* Mak (DOC)

S4 Table SSR primers used in the diversity analysis of different diospyros germplasms. (DOC)

S5 Table Genetic distances and geographical distances between natural diospyros populations in Guangxi. (DOC)

## References

[1] Yonemori K (1997) Diospyros industry and research activities in Japan. Acta Hort 436: 21–32.

[2] Du XY, Zhang MS, Luo ZR, Zhang QL (2009) Identification and GeneticRelationships of diospyros kakiThunb. and Related SpeciesUsing ISTR Analysis. Acta Horticulturae Sinica 36 (4): 481–486.

[3] Wang RZ, Yang Y, Li GC (1997) Chinese diospyros germplasm resources. Acta Hortic 436: 43–50.

[4] Guo DL, Luo ZR (2006) Genetic relationships of some PCNA persimmons (*Diospyros kaki* Thunb.) from China and Japan revealed by SRAP analysis. Genet Resour Crop Ev. 53: 1597–1603.

[5] Wang RZ (1987) Persimmmon. Chin For press, Beijing 619–636.

[6] Luo ZR, Cai LH, Hu CG (1996) Research development of persimon germplasm resources of *Diospyros kaki* and their Utilization. J. Huazhong Agric Univ. 15 (4): 381–388.

[7] FAO (2012) FAOSTAT Database. http://faostat.fao.org/

[8] Deng LB (2013) Research on the genetic Diversity and angular leaf spot disease resistance of persimmon germplasm resources in Guangxi. Doctoral dissertation of Guangxi univ. Nanning 15–25.

[9] Wang R, Yang Y, Li GC (1997) Chinese persimmon germplasmresources. Acta Hortic 436: 43–50.

[10] Yang Y, Wang RZ, Li GC, Wang W (1999) Study on Chromosome Numbers of diospyros and Their Varieties. Acta Agriculturae Boreali-occidentalis Sinica 8 (3): 64–67.

[11] Kim TC, Ko KC (1997) Taxonomic studies of persimmon (*Diospyros Kaki* Thumb.) by multivariate and isozyme analysis. Acta Horticulturae 436: 85–92.

[12] Luo ZR, Yonemori K, Sugiura A (1995) Evaluation of RAPD analysis for cultivar identification of persimmons (*Diospyros kaki*). Journal of the Japanese Society for Horticultural Science 64(3): 535–541.

[13] Naval MDM, Zuriaga E, Pecchioli S, Llácer G, Giordani E, Badenes ML (2010) Analysis of genetic diversity among persimmon cultivars using microsatellite markers. Tree Genet. Genomes 6: 677–687.

[14] Hu DC, Zhang QL, Luo ZR (2008) Phylogenetic analysis in some *Diospyros spp*. (Ebenaceae) and Japanese persimmon using chloroplast DNA PCR-RFLP markers. Scientia Horticulturae 117: 32–38.

[15] Yonemori K, Honsho C, Kitajima A, Aradhya M, Giordani E, Bellini E, et al (2008) Relationship of European persimmon (*Diospyros* kaki Thunb.) cultivars to Asian cultivars, characterized using AFLPs. Genet Resour Crop Evol 55: 81–89.

[16] Collard BCY, Mackill DJ (2009) Start codon targeted (SCoT) polymorphism: a simple, novel DNA marker technique for generating gene-targeted markers in plants. Plant Mol. Biol. Rep. 27: 86–93.

[17] Mulpuri S, Muddanuru T, Francis G (2013) Start codon targeted (SCoT) polymorphism in toxic and non-toxic accessions of Jatropha curcas L. and development of a codominant SCAR marker. Plan. Sci 207: 117–127. 10.1016/j.plantsci.2013.02.013 23602106

[18] Xiong FQ, Zhong RC, Han ZQ (2011) Start codon targeted polymorphism for evaluation of functional genetic variation and relationships in cultivated peanut (*Arachis hypogaea* L.) genotypes. Mol Biol Rep 38: 3487–3494. doi: 10.1007/s11033-010-0459-6 21104441 10.1007/s11033-010-0459-6

[19] Amirmoradi B, Talebi R, Karami E (2012) Comparison of genetic variation and differentiation among annual Cicer species using start codon targeted (SCoT) polymorphism, DAMD-PCR, and ISSR markers. Plant Syst Evol 298: 1679–1688.

[20] Doyle JJ, Doyle JL (1990) Isolation of plant DNA from fresh tissue. Focus 12:13–15.

[21] Soriano JM, Pecchioli S, Romero C, Vilanova S, Llácer G, Giordani E, et al (2006) Development of microsatellite markers in polyploid persimmon (*Diospyros* kaki Lf) from an enriched genomic library. Mol Ecol Notes 6: 368–370.

[22] He XH, Li YR, Guo YZ, Tang ZP, Li RB (2005) Genetic analysis of 23 mango cultivar collection in Guangxi province revealed by ISSR. Mol. Plant Breed. 3: 829–834.

[23] Guo DL, Luo ZR (2004) Establishment of SSR technique of *Diospyros kaki* and *D*, *lotus*[J]. Journal of Agricultural Biotechnology 12(4): 386–389.

[24] Zhang YF, Zhang QL, Yang Y, Luo ZR (2009) Deleopment of Japanese persimmon core collection by genetic distance sampling based on SSR markers. Biotechnol. & Biotechnol. Eq. 23(4): 1474–1478.

[25] Liang QZ, Wen DQ, Xie JH, Liu LQ, Wei YZ, Wang YC and Shi SY (2014) A rapid and effective method for silver staining of PCR products separated in polyacrylamide gels. Electrophoresis 35(17): 2520–2523. doi: 10.1002/elps.201400182 24789566 10.1002/elps.201400182

[26] Rohlf FJ (2000) NTSYS-pc: Numerical taxonomy and multivariate analysis system, version 2.1 Exeter Software, Setauket, New York.

[27] Sneath PHA, Sokal RR (1973) Numerical taxonomy. The principles and practice of numerical classification. pp. xv + 573 pp

[28] Peakall R, Smouse PE (2012) GenAlEx 6.5: genetic analysis in Excel. Population genetic software for teaching and research- an update. Bioinforma 28: 2537–2539. 10.1093/bioinformatics/bts460 PMC3463245 22820204

[29] Nei M (1972) Genetic distance between populations. Am Naturalist 106: 283–292.

[30] Gower JC (1966) Some distance properties of latent root and vector methods used in multivariate analysis. Biometrika 53: 325–338.

[31] Yang Y, Ruan XF, Wang RZ, Li GC (2005) A dvances in research of germplasm resources and breeding of Dispyros kaki L. J. Northwest For Univ 20: 133–137.

[32] Ai CX, Qin ZH, Tao JH, Wang CJ (2011) SSR fingerprints and genetic variations of the 32 persimmon major cultivars. Acta Bot. Borea1 3l: 2185–219l.

[33] Wu S, Fu JM, Wuyun TN, Liang YQ (2012) Study on EST-SSR primer design and genetic deversity of *Diospyros* L. J. Central South Univ. For. Tech. 32: 153–157.

[34] Deng LB, He XH, Li TW, Hu Y (2012) Investigation and analysis on the genetic diversity of diospyros germplasms in plateau of northwest Guangxi. Acta Hortic. Sin 39: 215–224.

[35] Gong BC, Wang RZ, Yang Y (2011) High quality practical technology of diospyros cultivation China For press, Beijing 67–68.

[36] Fischer M, Matthies D (1998) RAPD variation in relation to population size and plant fitness in the rare Gentianella germanica (*Gentianaceae*). Am. J. Bot. 85: 811–819. 21684965

[37] Ge XJ, Zhang LB, Yuan YM, Hao G (2005) Strong genetic differentiation of the East-Himalayan Megacodon stylophorus (*Gentianaceae*) detected by intersimple sequence repeats (ISSR). Biodivers Conserv. 14: 849–861

[38] Wright S (1951) The genetical structure of populations. Ann. Eug. 15: 323–354. 10.1111/j.1469-1809.1949.tb02451.x 24540312

[39] He XH, Pan H, Deng LB, Pan JC, Li F et al (2010) Genetic diversity of natural Myrica rubra Sieb.et Zucc populations in Guangxi revealed by ISSR markers. Sci. Agric. sin. 9: 626–632.

