# Supplementary figures and images for: Investigation and Analysis of Genetic Diversity of Diospyros Germplasms Using SCoT Molecular Markers in Guangxi
Source: PLoS One. 2015 Aug 28;10(8):e0136510. doi: 10.1371/journal.pone.0136510 (PMC4552666; doi:10.1371/journal.pone.0136510)

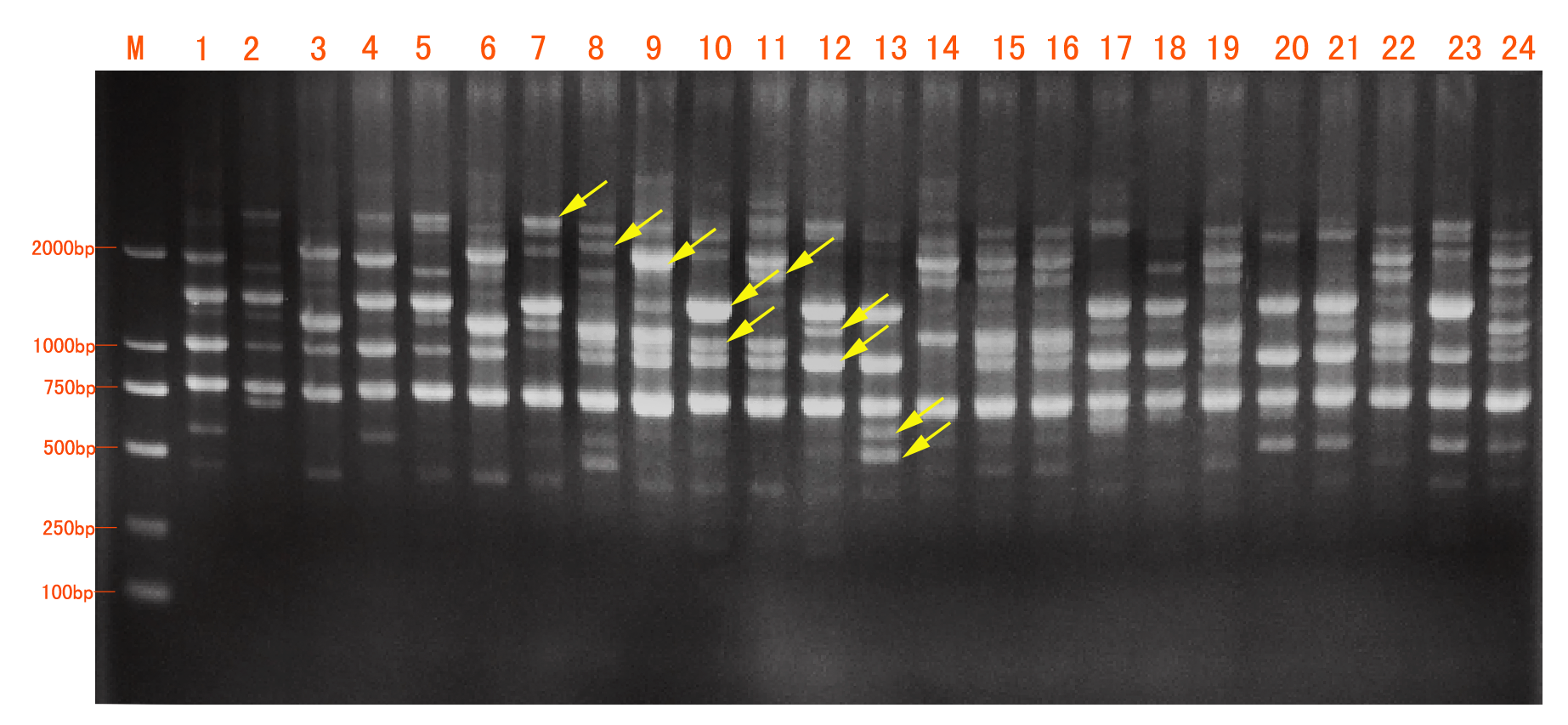

Supplement: S1 Fig — (TIF) [file pone.0136510.s001.tif]

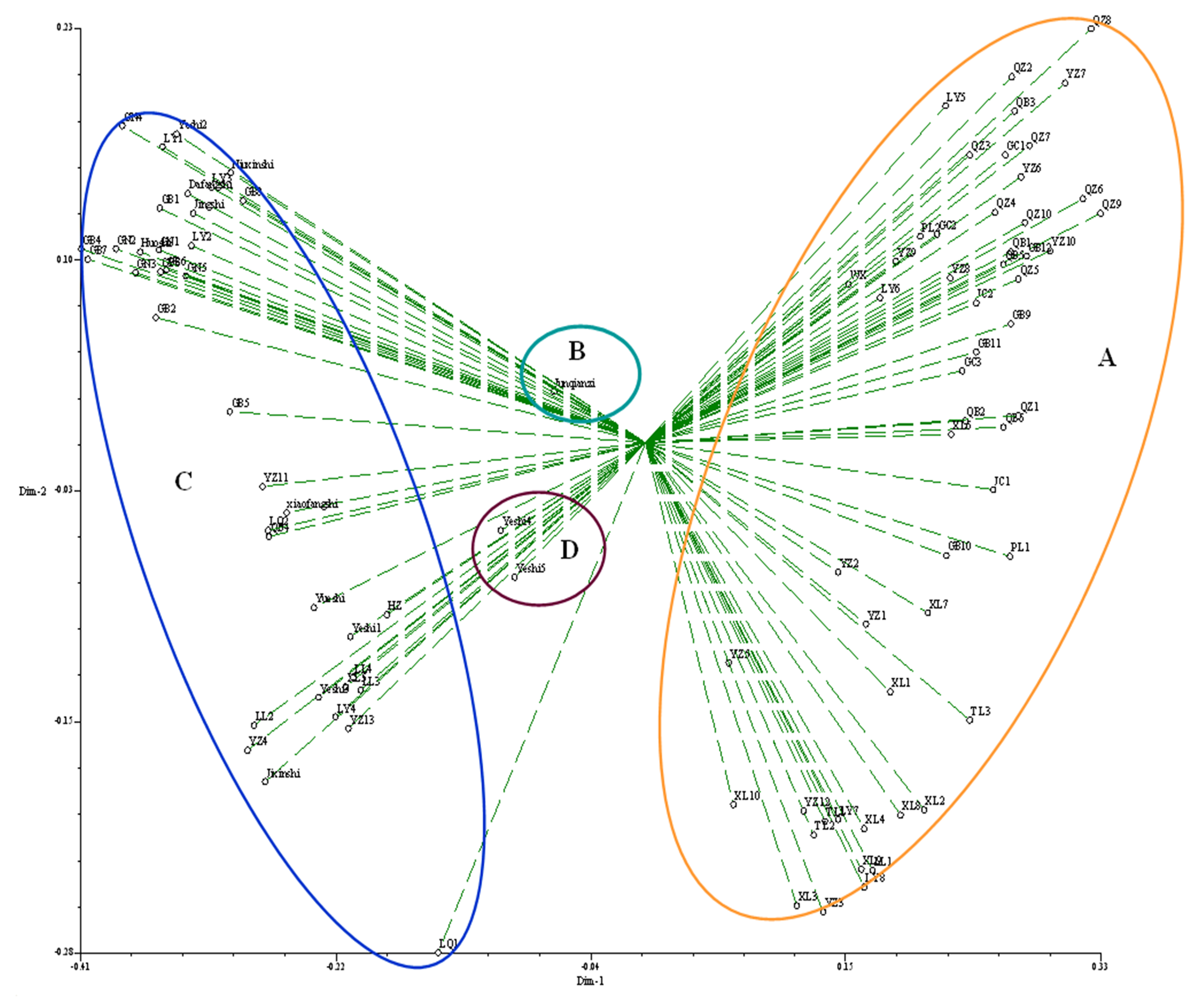

Supplement: S2 Fig — (TIF) [file pone.0136510.s002.tif]

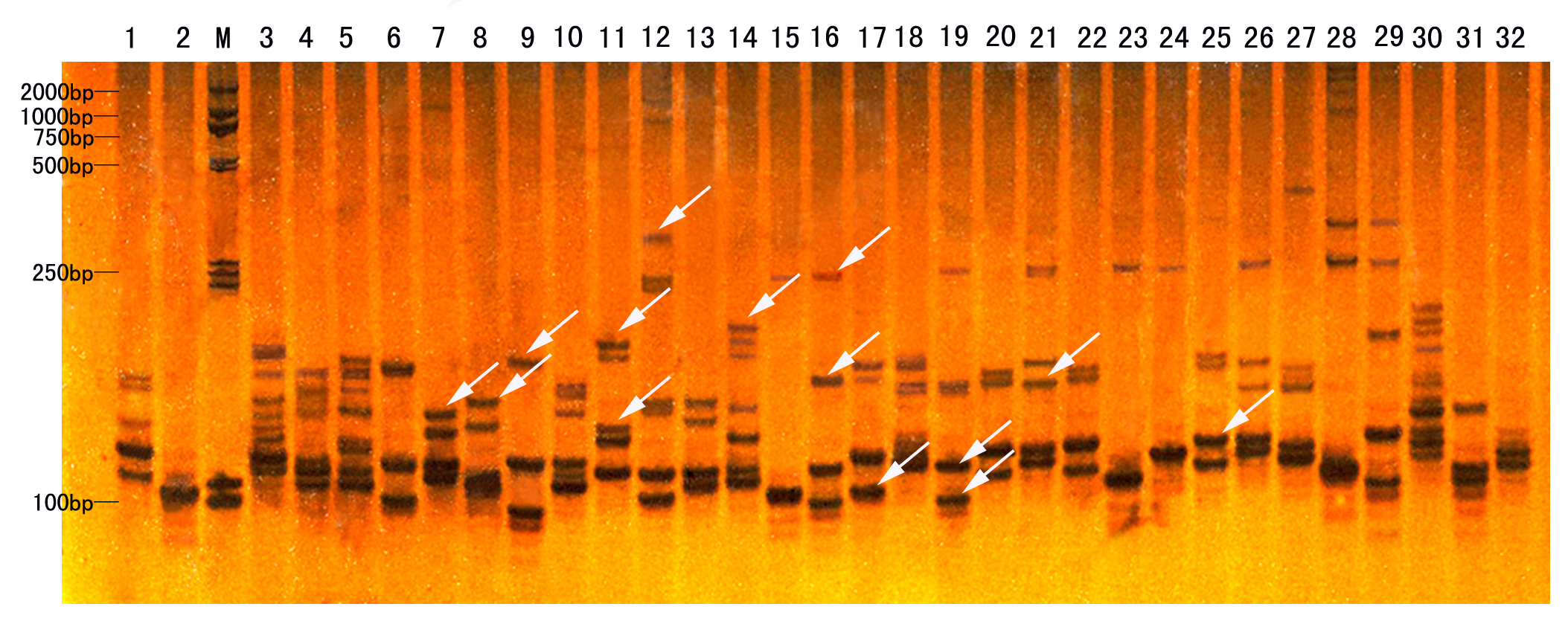

Supplement: S3 Fig — (TIF) [file pone.0136510.s003.tif]

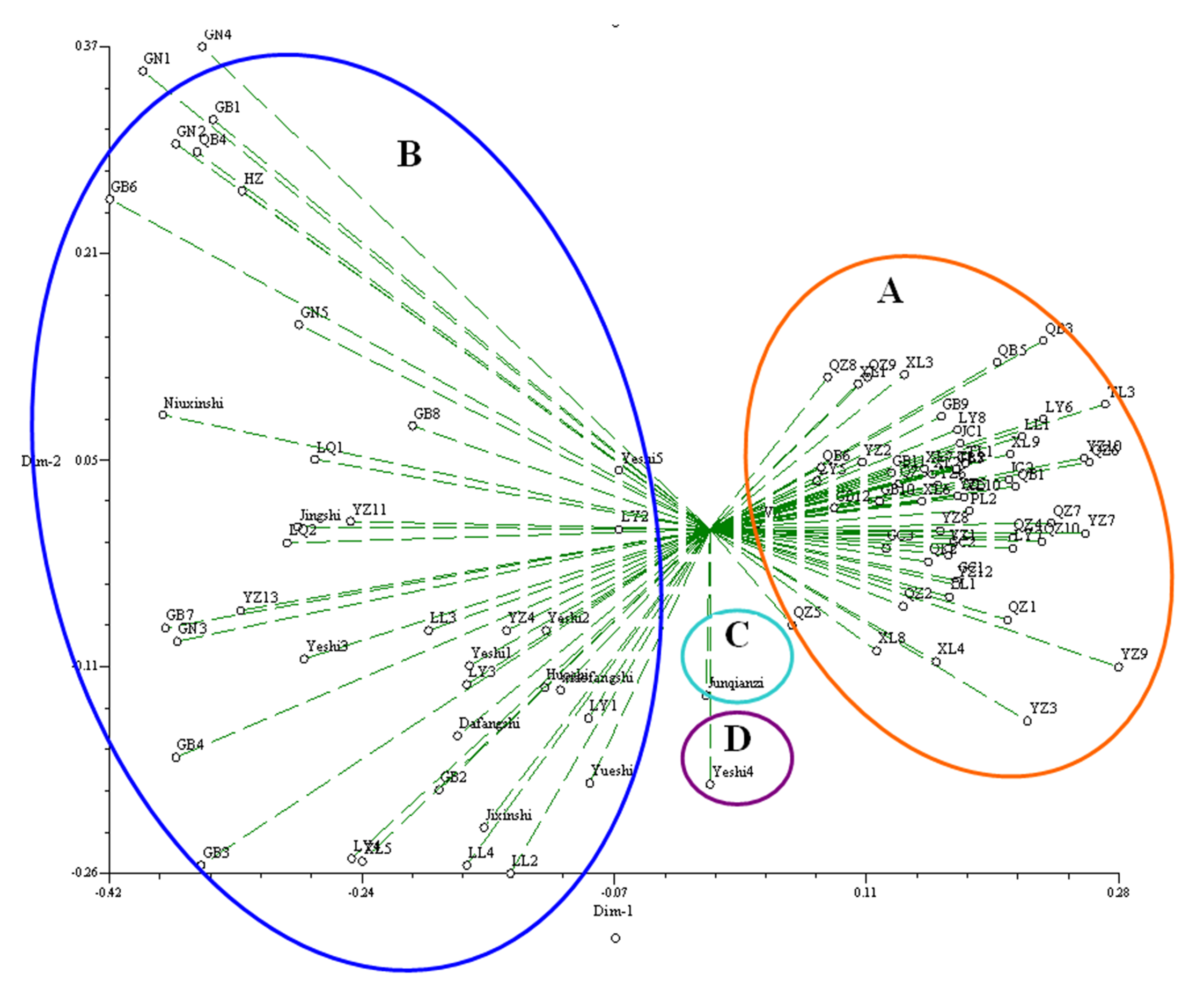

Supplement: S4 Fig — (TIF) [file pone.0136510.s004.tif]

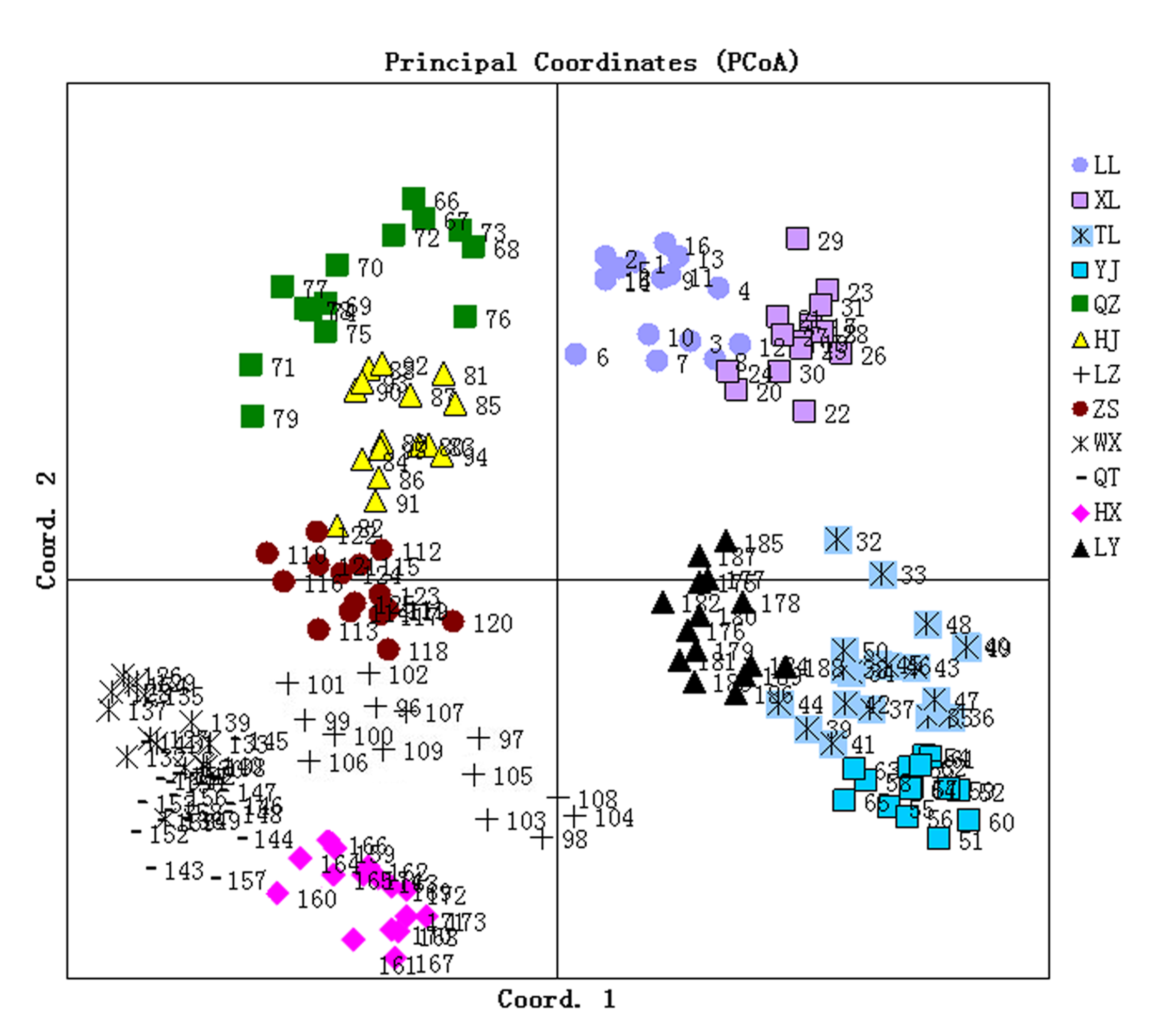

Supplement: S5 Fig — (TIF) [file pone.0136510.s005.tif]
