# Supplementary material for: Investigation and Analysis of Genetic Diversity of Diospyros Germplasms Using SCoT Molecular Markers in Guangxi
Source: PLoS One. 2015 Aug 28;10(8):e0136510. doi: 10.1371/journal.pone.0136510 (PMC4552666; doi:10.1371/journal.pone.0136510)
Supplement: S1 Table — (DOC) [file pone.0136510.s006.doc]

S1 Table. The surveyed populations and their respective ecological and geographical parameters.

| No | Populations | Localities | Longitude and Latitude | Altitude  (m) | Rain fall  (mm) | Sample size |
| --- | --- | --- | --- | --- | --- | --- |
| 1 | LL | Longlin county | 24°49'54.09"N 105°13'47.02"E | 1224 | 1218 | 15 |
| 2 | XL | Xilin county | 24°31'57.60" N 104°46'38.12"E | 1246 | 1700 | 16 |
| 3 | TL | Tianlin county | 24°13'42.33" N 106°11'45.42"E | 479 | 1550 | 15 |
| 4 | YJ | Youjian county | 23°50'31.19" N 106°23'58.89"E | 332 | 1115 | 19 |
| 5 | QZ | Quanzhou county | 25°49'33.58" N 110°44'12.49"E | 565 | 1519 | 14 |
| 6 | HJ | Huanjian county | 25°09'34.82" N 108°36'28.81"E | 486 | 1389 | 16 |
| 7 | ZS | Zhongshan county | 24°07'35.55" N 111°18'57.86"E | 150 | 1550 | 14 |
| 8 | LZ | Luzhai county | 24°32'07.01" N 109°34'58.46"E | 214 | 1500 | 16 |
| 9 | WX | Wuxuan county | 23°36'53.86" N 109°46'52.72"E | 103 | 1300 | 15 |
| 10 | QT | Qintang county | 23°12'46.17" N 109°31'25.09"E | 67 | 1325 | 18 |
| 11 | HX | Hengxian county | 22°56'02.64" N 108°57'20.52"E | 52 | 1685 | 16 |
| 12 | LY | Leye county | 25°42'29.88"N 106°20'23.00"E | 1501 | 1350 | 15 |
| total |  |  |  |  |  | 189 |
