# Supplementary material for: Investigation and Analysis of Genetic Diversity of Diospyros Germplasms Using SCoT Molecular Markers in Guangxi
Source: PLoS One. 2015 Aug 28;10(8):e0136510. doi: 10.1371/journal.pone.0136510 (PMC4552666; doi:10.1371/journal.pone.0136510)
Supplement: S2 Table — (DOC) [file pone.0136510.s007.doc]

S2 Table. SCoT primers used in the diversity analysis of different diospyros germplasms.

| Primers | Sequences (5'-3') | TNB^a^ | NPB^b^ | Polymorphic ratio (%) |
| --- | --- | --- | --- | --- |
| SCoT7 | CAACAATGGCTACCACGG | 15 | 14 | 93.33 |
| SCoT10 | CAACAATGGCTACCAGCC | 14 | 14 | 100.00 |
| SCoT12 | ACGACATGGCGACCAACG | 15 | 14 | 93.33 |
| SCoT16 | ACCATGGCTACCACCGAC | 17 | 17 | 100.00 |
| SCoT17 | CAACAATGGCTACCACGG | 12 | 11 | 91.67 |
| SCoT19 | ACCATGGCTACCACCGGC | 11 | 11 | 100.00 |
| SCoT20 | ACCATGGCTACCACCGCG | 14 | 14 | 100.00 |
| SCoT21 | ACGACATGGCGACCCACA | 11 | 10 | 90.91 |
| SCoT29 | CCATGGCTACCACCGGCC | 13 | 13 | 100.00 |
| SCoT30 | CCATGGCTACCACCGGCG | 12 | 12 | 100.00 |
| SCoT32 | CCATGGCTACCACCGCAC | 16 | 16 | 100.00 |
| SCoT44 | CAATGGCTACCATTAGCC | 12 | 12 | 100.00 |
| SCoT52 | ACAATGGCTACCACTGCA | 9 | 7 | 77.78 |
| SCoT55 | ACAATGGCTACCACTACC | 12 | 11 | 91.67 |
| SCoT60 | ACAATGGCTACCACCACA | 16 | 16 | 100.00 |
| SCoT61 | CAACAATGGCTACCACCG | 10 | 10 | 100.00 |
| SCoT73 | CCATGGCTACCACCGGCT | 15 | 15 | 100.00 |
| SCoT74 | CCATGGCTACCACCGGCA | 17 | 16 | 94.12 |
|  |  | 241 | 233 | 96.68 |

Note：TNB^a^ =Total number of bands；NPB^b^=Number of polymorphic bands.
