# Supplementary material for: Investigation and Analysis of Genetic Diversity of Diospyros Germplasms Using SCoT Molecular Markers in Guangxi
Source: PLoS One. 2015 Aug 28;10(8):e0136510. doi: 10.1371/journal.pone.0136510 (PMC4552666; doi:10.1371/journal.pone.0136510)
Supplement: S3 Table — (DOC) [file pone.0136510.s008.doc]

S3 Table. SCoT primers used in the diversity analysis of natural populations of *D. kaki var. silverstris* Mak.

| Primers | Sequences(5'-3') | TNB^a^ | NPB^b^ | Polymorphic ratio (%) |
| --- | --- | --- | --- | --- |
| SCoT7 | CAACAATGGCTACCACGG | 15 | 14 | 93.33 |
| SCoT8 | CAACAATGGCTACCACGT | 7 | 7 | 100.00 |
| SCoT10 | CAACAATGGCTACCAGCC | 11 | 11 | 100.00 |
| SCoT12 | ACGACATGGCGACCAACG | 14 | 14 | 100.00 |
| SCoT20 | ACCATGGCTACCACCGCG | 13 | 13 | 100.00 |
| SCoT21 | ACGACATGGCGACCCACA | 8 | 7 | 87.50 |
| SCoT30 | CCATGGCTACCACCGGCG | 16 | 16 | 100.00 |
| SCoT33 | CAATGGCTACCACCGACG | 12 | 12 | 100.00 |
| SCoT44 | CAATGGCTACCATTAGCC | 12 | 12 | 100.00 |
| SCoT49 | ACAATGGCTACCACTACC | 11 | 11 | 100.00 |
| SCoT55 | ACAATGGCTACCACTACC | 12 | 12 | 100.00 |
| SCoT73 | CCATGGCTACCACCGGCT | 14 | 12 | 85.71 |
| SCoT74 | CCATGGCTACCACCGGCA | 14 | 14 | 100.00 |
|  |  | 159 | 155 | 97.48 |

Note：TNB^a^ =Total number of bands；NPB^b^=Number of polymorphic bands.
