# Supplementary material for: Investigation and Analysis of Genetic Diversity of Diospyros Germplasms Using SCoT Molecular Markers in Guangxi
Source: PLoS One. 2015 Aug 28;10(8):e0136510. doi: 10.1371/journal.pone.0136510 (PMC4552666; doi:10.1371/journal.pone.0136510)
Supplement: S4 Table — (DOC) [file pone.0136510.s009.doc]

S4 Table. SSR primers used in the diversity analysis of different diospyros germplasms.

| Primer name | Primer sequence (5'-3') | Size of amplified  band | Total amplifying  bands | Polymorphic  bands | Polymorphic  ratio |
| --- | --- | --- | --- | --- | --- |
| SSR19 | F:CTAAATCCCCCTTTCTTCAT  R:TAGTCGCCTTCGTCTCCTCC | 110～190 bp | 9 | 8 | 88.89 |
| SSR27 | F:GGCATTCCCCCTTTCTTCAT  R:TTTTTCATCCATGCCACTGA | 120～190 bp | 8 | 8 | 100.00 |
| SSR29 | F:AGATGGAGTGACAGCAGAGACTG  R:CCCCTTAAGTCTTAGCTAATTAC | 130～200 bp | 9 | 9 | 100.00 |
| SSR30 | F:GTAATTAGCTAAGACTTAAGGGG  R:TGCTACAACAACTGGAAGAC | 110～190 bp | 7 | 6 | 85.71 |
| SSR31 | F:GTGAAGGAACCCCTAAGAA  R:CCATCATCAGGTAGGAGAGA | 150～250 bp | 12 | 12 | 100.00 |
| SSR32 | F:ACTACAACGGCGGTGAGAAC  GTCCTTCACTTCTTCCCGCATT | 110～260 bp | 14 | 14 | 100.00 |
| SSR33 | F:GCTTCGGGTATCCACACTTC  R:CACTGTGAGCGATTTCTTCG | 100～500 bp | 11 | 11 | 100.00 |
| SSR34 | F:ATGAGAGAGAGAGAATGATTGATGC  R:CATTTTGCACGCAGTGAGAT | 220～280 bp | 13 | 12 | 92.31 |
| SSR39 | F:CGAGCAAGTAGATGTTTATT  R:TCATGATGATTAAAGAGGAC | 120～260 bp | 11 | 11 | 100.00 |
| SSR40 | F:ATCATGAGATCAGAGCCGTC  R:CACGTTAACGTTACGGAACA | 80～300 bp | 14 | 14 | 100.00 |
| SSR42 | F:AGTTCTTGCGATGGGATTTG  R:GATGAGATGGGCTGATTGCT | 130～400 bp | 11 | 10 | 90.91 |
| SSR44 | F:GGGAAGAACAAAGAGAACTG  R:ACGAAGTTGTAATCCTGAGC | 200～320 bp | 9 | 8 | 88.89 |
| SSR46 | F:ACACAGGCAGACAAATTCATTC  R:CCATAGGCATTGCTGCCATT | 90～210 bp | 10 | 10 | 100.00 |
| SSR48 | F:ACACCCCTTCTTTTATAC  R:ATCCAGGAGGGCAAAGAACT | 80～250 bp | 11 | 11 | 100.00 |
| SSR53 | F:AGAAGACCCAGACCAGAGAAC  R:GGCACACAAATCAACCATACC | 90～210 bp | 10 | 10 | 100.00 |
|  | Total |  | 159 | 154 |  |
