# Supplementary material for: Investigation and Analysis of Genetic Diversity of Diospyros Germplasms Using SCoT Molecular Markers in Guangxi
Source: PLoS One. 2015 Aug 28;10(8):e0136510. doi: 10.1371/journal.pone.0136510 (PMC4552666; doi:10.1371/journal.pone.0136510)
Supplement: S5 Table — (DOC) [file pone.0136510.s010.doc]

S5 Table. Genetic distances and geographical distances between natural diospyros populations in Guangxi.

| Populations | LL | XL | TL | YJ | QZ | HJ | ZS | LZ | WX | QT | HX | LY |
| --- | --- | --- | --- | --- | --- | --- | --- | --- | --- | --- | --- | --- |
| LL |  | 69.89 | 115.56 | 160.79 | 572.13 | 338.26 | 613.42 | 442.9 | 480.87 | 471.65 | 236.43 | 183.31 |
| XL | 0.11 |  | 148.65 | 182.91 | 663.02 | 393.36 | 658.25 | 497.28 | 524.04 | 507.91 | 251.21 | 243.87 |
| TL | 0.166 | 0.104 |  | 45.95 | 513.26 | 268.91 | 517.91 | 356.12 | 379.50 | 361.11 | 173.63 | 134.03 |
| YJ | 0.236 | 0.195 | 0.101 |  | 510.13 | 267.33 | 494.17 | 339.29 | 352.91 | 330.30 | 158.85 | 153.69 |
| QZ | 0.138 | 0.199 | 0.215 | 0.292 |  | 224.53 | 219.78 | 203.41 | 289.05 | 336.34 | 203.19 | 392.71 |
| HJ | 0.179 | 0.196 | 0.207 | 0.273 | 0.156 |  | 294.30 | 128.06 | 211.05 | 233.54 | 129.30 | 155.16 |
| ZS | 0.186 | 0.217 | 0.158 | 0.207 | 0.183 | 0.175 |  | 170.71 | 156.62 | 199.31 | 147.77 | 435.47 |
| LZ | 0.174 | 0.19 | 0.187 | 0.235 | 0.186 | 0.16 | 0.139 |  | 106.94 | 146.34 | 103.44 | 264.66 |
| WX | 0.208 | 0.215 | 0.223 | 0.253 | 0.175 | 0.165 | 0.161 | 0.137 |  | 42.71 | 62.80 | 319.94 |
| QT | 0.218 | 0.227 | 0.189 | 0.244 | 0.185 | 0.168 | 0.149 | 0.137 | 0.091 |  | 44.33 | 323.04 |
| HX | 0.164 | 0.15 | 0.11 | 0.159 | 0.17 | 0.15 | 0.154 | 0.129 | 0.12 | 0.112 |  | 162.55 |
| LY | 0.214 | 0.174 | 0.167 | 0.198 | 0.248 | 0.217 | 0.182 | 0.189 | 0.231 | 0.198 | 0.175 |  |

Note：Geographical distance in kilometers is above the diagonal and genetic distance is below.
